# Morphology and distribution of taste papillae and oral denticles in the developing oropharyngeal cavity of the bamboo shark, *Chiloscyllium punctatum*

**DOI:** 10.1242/bio.022327

**Published:** 2016-10-26

**Authors:** Carla J. L. Atkinson, Kyle J. Martin, Gareth J. Fraser, Shaun P. Collin

**Affiliations:** 1 School of Biomedical Sciences and the Queensland Brain Institute, The University of Queensland, St Lucia, Queensland 4072, Australia; 2 Department of Animal and Plant Sciences, University of Sheffield, Sheffield S10 2TN, UK; 3 The School of Animal Biology and the UWA Oceans Institute, The University of Western Australia, Crawley, Western Australia 6009, Australia

**Keywords:** Elasmobranch, Oral denticle, Taste, Taste buds, Taste papillae

## Abstract

Gustation in sharks is not well understood, especially within species that ingest food items using suction. This study examines the morphological and immunohistochemical characterisation of taste papillae and oral denticles in the oropharynx of the brown-banded bamboo shark *Chiloscyllium punctatum* and compares their distribution during development. Taste papillae of *C. punctatum* are located throughout the oropharyngeal region and are most concentrated on the oral valves (2125-3483 per cm^2^ in embryos; 89-111 per cm^2^ in mature adults) close to the tooth territories. Papillae appearance is comparable at all stages of development, with the exception of the embryos (unhatched specimens), where no microvilli are present. Oral valve papillae are comparable in structure to Type I taste buds of teleost fishes, whereas those of the rest of the oropharyngeal region are comparable to Type II. Both types of papillae show immunofluorescence for a number of markers of taste buds, including β-Catenin and Sox2. Taste papillae densities are highest in embryos with 420-941 per cm^2^ compared to 8-29 per cm^2^ in mature adults. The total number of papillae remains around 1900 for all stages of development. However, the papillae increase in diameter from 72±1 μm (mean±s.e.m.) in embryos to 310±7 μm in mature individuals. Microvilli protrude in multiple patches at the apical tip of the papilla covering ∼0.5% of the papillar surface area. We further document the relationship between taste papillae and the closely associated oral denticles within the shark orophayngeal cavity. Oral denticles first break through the epithelium in the antero-central region of the dorsal oral cavity, shortly after the emergence of teeth, around time of hatching. Denticles are located throughout the oropharyngeal epithelium of both immature and mature stages, with the highest concentrations in the antero-dorsal oral cavity and the central regions of the pharynx. These denticle-rich areas of the mouth and pharynx are therefore thought to protect the epithelium, and importantly the taste papillae, from abrasion since they correlate with regions where potential food items are processed or masticated for consumption. Taste papillae and denticles are more dense in anterior oropharyngeal regions in close association with the oral jaws and teeth, and in the juvenile or hatchling shark taste units are functional, and innervated, allowing the shark to seek out food *in utero*, at birth or on emergence from the egg case.

## Abstract (precis)

**Summary:** Characterisation of taste buds in the developing bamboo shark, *Chiloscyllium punctatum*, reveals that taste papillae are functional, innervated units, allowing the shark to seek out food *in utero*, at birth or following hatching.

## INTRODUCTION

Taste buds are secondary sense organs of the gustatory chemosensory system involved in the evaluation of food quality. Taste buds in teleost fish are more numerous than in any other animal (Kasumyan and Døving, 2003), yet they are poorly understood. Within teleost fishes, they are present on the surface of the skin, lips, fins and barbels as well as within the epithelia of the oral cavity, pharynx, oesophagus and gills (Jakubowski and Whitear, 1990; Reutter et al., 2000). Their broad distribution distinguishes teleosts from other gnathostomes, which only contain taste buds within the oral cavity. An exception is the Amphibia, which possess taste buds on the skin of the head at some developmental stages (Kasumyan and Døving, 2003). Gustation is a contact sense and therefore aquatic organisms differ from terrestrial organisms, as the medium in which they live is a constant vector of chemical stimuli. Three types of taste buds exist in teleosts; Type I protrude the furthest above the surrounding epithelium and have a depression around their base, which is lower than the surrounding epithelium. Type II are similar to Type I but lack the depression and Type III occur within a pore on the flat cornified, desquamating epithelium (Reutter et al., 1974). Taste buds comprise receptor cells, support cells and sometimes basal cells and are innervated by branches of the VII (facial), IX (glossopharyngeal) and X (vagal) cranial nerves (Reutter, 1992).

There is a great paucity of literature on elasmobranch gustation with no record of any ontogenetic differences in either the density or distribution of taste papillae within the oropharyngeal cavity or over associated structures. The external skin of elasmobranchs is covered with protective scales known as placoid scales or denticles (Kemp, 1999), which are composed of a calcified base and dentine protrusion covered by an enamel cap (Granvendeel et al., 2002). Denticles are also present in the oral mucosa (Hertwig, 1874; Steinhard, 1903; Imms, 1905) and appear to have evolved a structure used for altering hydrodynamic flow over the gills during swimming (especially in those species that are required to maintain forward movement or a method of breathing known as ram ventilation) or for protection from abrasion (Atkinson and Collin, 2012). The density and distribution of denticles appears to compromise that of the taste papillae as each compete for space (Atkinson and Collin, 2012).

In this study, light microscopy, immunohistochemistry and both scanning and transmission electron microscopy are used to characterize the different types of taste papillae and determine whether there are ontogenetic changes in the structure and distribution of the taste papillae and oral denticles in the brown-banded bamboo shark, *Chiloscyllium punctatum* Muller and Henle 1838. *Chiloscyllium* *punctatum* is a relatively common benthic selachian found off the southeast coast of Queensland, Australia, and the subject of a captive breeding program at UnderWater World, on the Sunshine Coast. This access to an important model species of elasmobranch provided a range of developmental stages of both wild-caught and captive-bred individuals. *Chiloscyllium* *punctatum* is found in coral reefs, tidal pools, sea grass beds and mangrove bays (Last and Stevens, 1994) and is a benthic suction feeder (Lowry and Motta, 2008; Wilga and Sanford, 2008; Goto et al., 2013). This involves ingesting prey by moving fluid rapidly into the oral cavity by increasing the pressure differential between the inside of the mouth and the surrounding environment following buccal expansion. This has been found to be variable in teleosts with the size of the mouth aperture, the morphology of the jaw and strike behaviour all contributing to the effectiveness of this type of feeding (Lauder, 1980; Van Leeuwin, 1984; Wainwright et al., 2007). It feeds on, in descending order of preference, annelid polychaete worms, crustaceans, teleost fishes and cephalopods. When comparing size classes, however, more teleost fishes and fewer annelid polychaete worms are consumed with increased body size [1000-1240 mm total length (TL)] with annelid polychaete worms dominating the stomach analyses of smaller individuals (400-740 mm TL) (Stead, 2008).

Our findings reveal that taste papillae appear early in development, closely linked with the timing of tooth development (Rasch et al., 2016) and we show that these taste buds are functional during later stages of embryo development before hatching. Densities of taste papillae were greatest in oral regions associated with the dentition and we suggest that there is a relationship between prey contact (jaws and teeth) and taste papillae density. Oral denticles, however, develop later during post-hatching ontogeny suggesting a shift toward active prey capture that includes both an indirect and direct abrasive diet, providing protection to the sensitive taste papillae within the oropharyngeal cavity of the bamboo shark.

## RESULTS

### Feeding observations

Feeding observations of *C. punctatum* reveal that they are suction feeders. Animals search for food items with their heads down against the substrate working in a sweeping motion to cover a large area. When a potential food item is found, the animal will inhale it head first and then rise up onto its pectoral fins, thereby elevating the head. This type of benthic suction feeding has also been observed in a number of other *Chiloscyllium* sp. (Lowry and Motta, 2008; Wilga and Sanford, 2008), where the internal movement of parts of the cranium and pressure in the buccal, hyoid and pharyngeal cavities generates a sequential change in suction pressure as prey is drawn into the mouth (Wilga and Sanford, 2008). A chomping action then ensues and small pieces of tissue may fall from the gill arches confirming the item is being crushed and possibly shredded. The jaws remain closed during this process, which appears to take place in the pharynx. Once the item has been consumed, the animal will remain still, whilst apparently ‘gasping’ excess amounts of water. The movement is more exaggerated than the normal buccal pumping respiration that takes place when not feeding and may help with the swallowing process, enabling the animal to open the oesophagus for easier passage of food to the stomach. Small hatchlings (*n*=15) also ingest food in this way, although due to the small size of their mouth items of food are more commonly spat out and re-consumed multiple times (up to three times), where each food item appears to be progressively more shredded. Food items considered too large to ingest whole are held in the jaws prior to a series of head-shakes to break up the item into smaller pieces. Greater suction pressures may be expected in larger individuals (as found for *C. plagiosum*, Lowry and Motta, 2008) but suction feeding occurs throughout ontogeny, where larger individuals would consume larger prey items.

### Taste papillae distribution

Taste papillae were distributed throughout the oropharyngeal epithelium with individual papillae oriented towards the centre of the oral and pharyngeal cavities. Papillae were only rarely found in the spiracles or on the receding gill arch epithelia and so these regions were discounted from the statistical analyses.

Data were square root- and log-transformed before analysis, in order to achieve approximate normality and homogeneity of variances. An ANCOVA was carried out to determine taste bud density differences between regions as the categorical variable with the developmental stage of the animal used as a covariate. Normality of the residuals was checked using a normal quantile-quantile plot and homogeneity of variances was checked by examining a plot of the residuals versus the fitted values from the model. No significant difference among slopes was found (*F*_11,216_=0.723, *P*=0.867), but there were significant differences between taste papillae densities in different regions (*F*_11,216_=42.150, *P*≤0.001). Tukey's pairwise comparisons were then performed to determine where these significant differences were located. Any differences mentioned are significant at the 5% significance level. Please refer to Figs S1-S4 for the mean densities±s.e.m. of papillae from each developmental stage as they are omitted from this text for clarity.

The density of taste buds in the maxillary and mandibulary valves were not different, but each has more taste buds than all other regions. Taste bud densities in central regions show no differences between each other or most side regions with the exception of the ventral central pharynx which has fewer taste buds than the sides of the oral cavity and the dorsal central pharynx which has fewer taste buds than the sides of the dorsal cavity. Central regions also have fewer taste buds than the anterior regions of the oral cavity. Taste bud densities in side regions are not different from each other with the exception of the dorsal oral cavity and pharynx, where the oral cavity has fewer taste buds.

In summary, no significant differences were found between the total numbers of taste papillae at the different stages of development or in individuals of different total length. The mean total number of taste papillae in *C. punctatum* is 1851±86. For all ontogenetic stages, taste bud densities in anterior regions are not different to the densities along the sides of the oral cavity but the anterior regions do have more taste buds than all central regions and the sides of the pharynx. The oral valves have the highest taste bud densities followed by the anterior regions of the oral cavity. Few significant differences are seen between the other regions.

### Taste papillae size

**Fig. 1.**
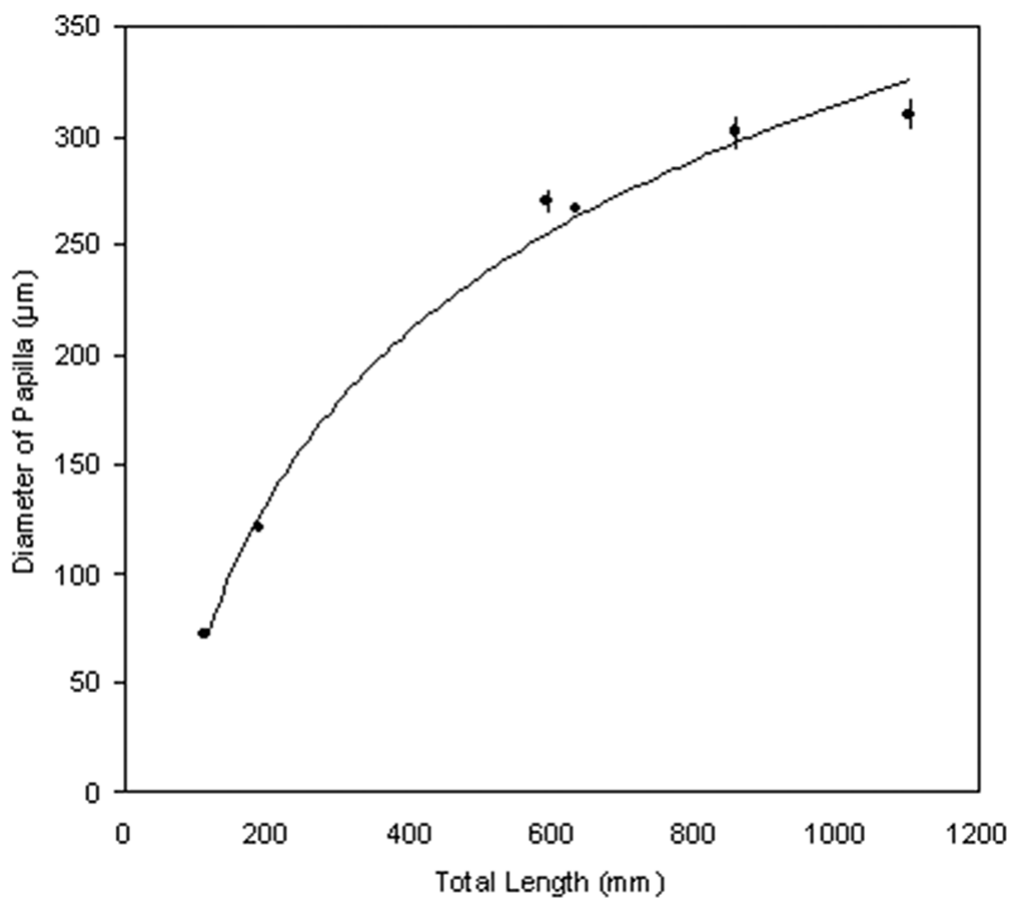
**Taste papillae size changes with growth.** Taste papillae diameters for various individuals of *Chiloscyllium punctatum* with different total lengths. Values are mean±s.e.m.

As the total length of the animal increases, so does the diameter of the taste papillae (Fig. 1). The smallest papillae measured in the present study were from an embryo (TL 116 mm, *n*=142) with a mean diameter of 72±1 μm. The largest papillae were from a mature individual (TL 1103 mm, *n*=27) with a mean diameter of 310±7 μm.

### Light microscopy and scanning electron microscopy of taste papillae

**Fig. 2.**
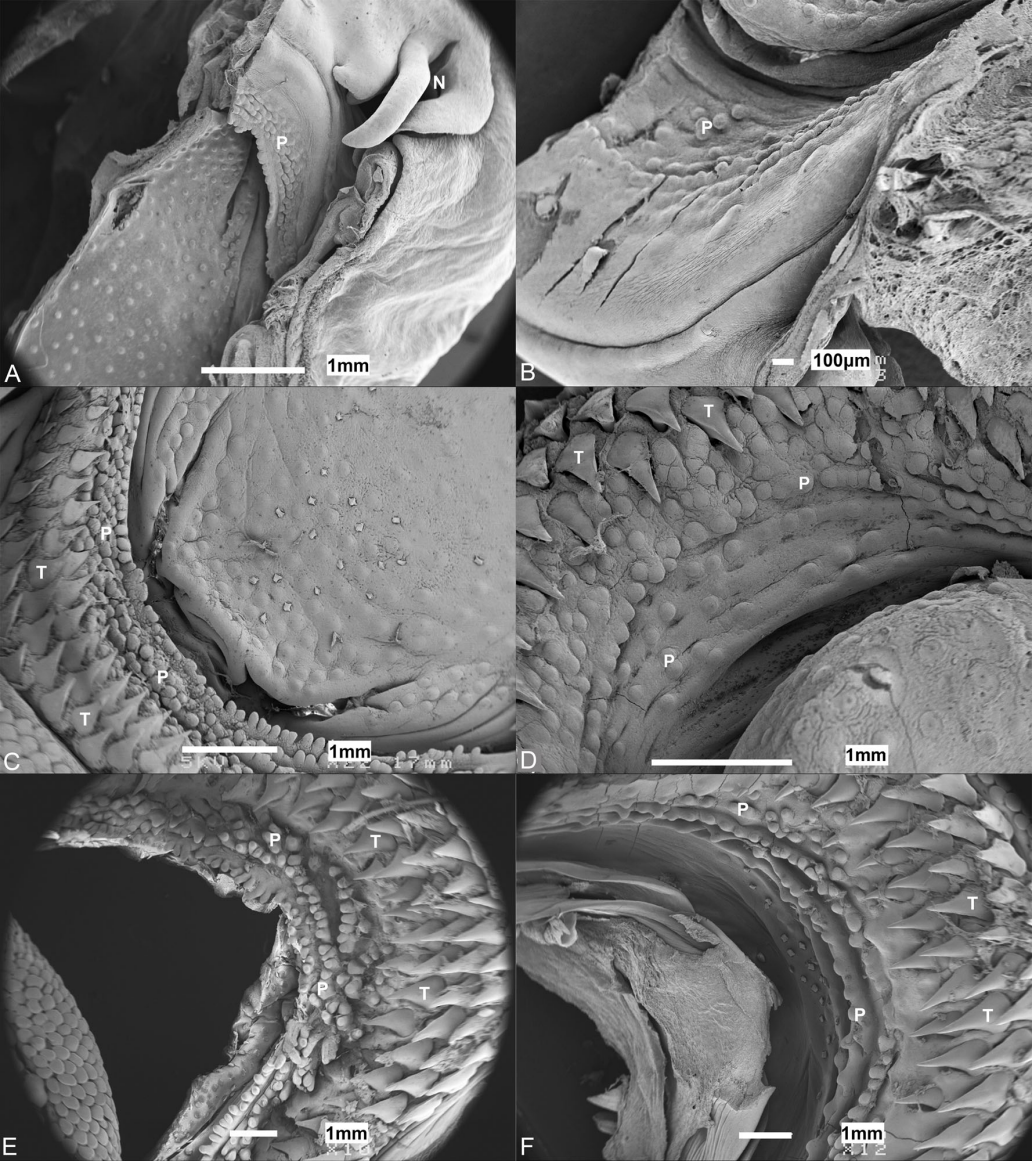
**Morphology of taste papillae.** Scanning electron micrographs of taste papillae (P) on the (A,C,E) maxillary and (B,D,F) mandibulary valves of an (A,B) embryo (TL 116 mm; note no teeth protruding), (C,D) hatchling (TL 192 mm), and (E,F) immature (TL 484 mm) *Chiloscyllium punctatum*. N, nare; T, teeth.

**Fig. 3.**
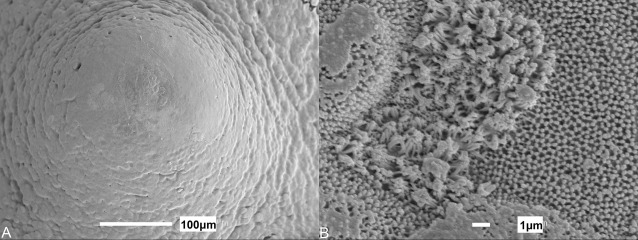
**Morphology of dorsal papillae in immature juveniles.** Scanning electron micrographs of (A) a taste papilla from the anterior dorsal oral cavity of *Chiloscyllium punctatum* (TL 635 mm), and (B) a magnified view of the microvilli protruding from the apical surface of a papilla.

The oral cavity comprises a mosaic-like pavement of pentagonal and hexagonal stratified squamous epithelial cells of ∼10 µm diameter. The surface structure of these cells was constructed of a dense pattern of microvilli and some microplicae or ridge-like folds of the surface of the epithelial cells (Andrews, 1976; Collin and Collin, 2000). Both maxillary and mandibulary valves are crescent-shaped, have an undulating surface, and taper at their edges. The mandibulary valve (Fig. 2B,D,F) has a less prominent margin to that of the maxillary valve (Fig. 2A,C,F), which is covered in projections. Observations at the level of the scanning electron microscope suggest these undulations and their associated projections are taste papillae, which appear as a succession of mounds, not always clearly distinguishable from each other. Throughout the oral cavity and pharynx, individual mounds (Fig. 3A), which protrude a small distance above the surrounding flat epithelium, are evenly distributed. In all stages of development, these mounds are more numerous on the oral valves than on any other region of the oral cavity or pharynx. The papillae over the maxillary valve protrude a larger distance above the surface of the surrounding epithelia than those of the mandibulary valve, and often occur in rows of finger-like projections, unlike the random distribution seen on the mandibulary valve.

**Fig. 4.**
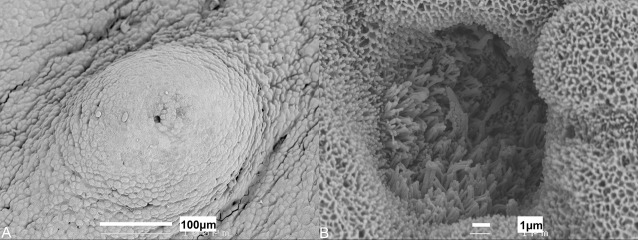
**Morphology of ventral papillae in immature juveniles.** Scanning electron micrographs of a taste papilla situated within the ventral oral cavity of *Chiloscyllium punctatum* (TL 635 mm) with both (A) protruding microvilli as well as (B) microvilli located within a pore.

**Fig. 5.**
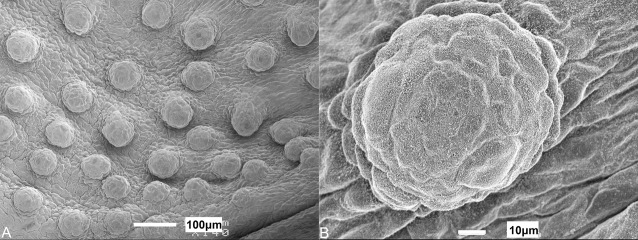
**Morphology of papillae in embryo.** Scanning electron micrographs of (A) taste papillae from an embryo *Chiloscyllium punctatum* (TL 116 mm). (B) Higher magnification of an individual papilla with no noticeable differentiated cells or microvilli protruding from the tip.

Microvilli (Fig. 3B) protrude above the surface of the epithelium at the apical tip of the papilla in patches that cover ∼0.5% of the surface area. Some papillae also have microvilli located within depressions in the apical surface (Fig. 4). Both groups of microvilli (those in depressions and those that protrude above the epithelial surface) may be found on papillae of the oral cavity and pharynx, and may even occur on the same individual papilla. Although this was seen for numerous papillae, it should not be discounted as an artefact of tissue preparation. The appearance of the papillae at all stages of development was comparable with the exception of the embryo stage in which no microvilli could be clearly identified on the papillae (Fig. 5). It is, however, important to note that even in mature individuals, not every papilla viewed using scanning electron microscopy would have such easily defined microvilli as those pictured (Figs 3 and 4), which may be the result of tissue preparation or reflect various stages of taste bud development, possibly with the depressions/pits representing degenerated taste cells (Beidler and Smallman, 1965).

It is important to note that serial sections (4 μm thick) of the oral papillae revealed slightly lighter stained cells in the central region but they did not reveal any microvillus cells protruding above the surface, nor did they form the stereotypical pear-shaped structure that has been previously reported. However, this issue has been previously noted for elasmobranch species (Reutter, 1993) and our molecular characterisation analyses confirm these structures are taste buds.

### Molecular characterization of the embryonic taste bud papillae

**Fig. 6.**
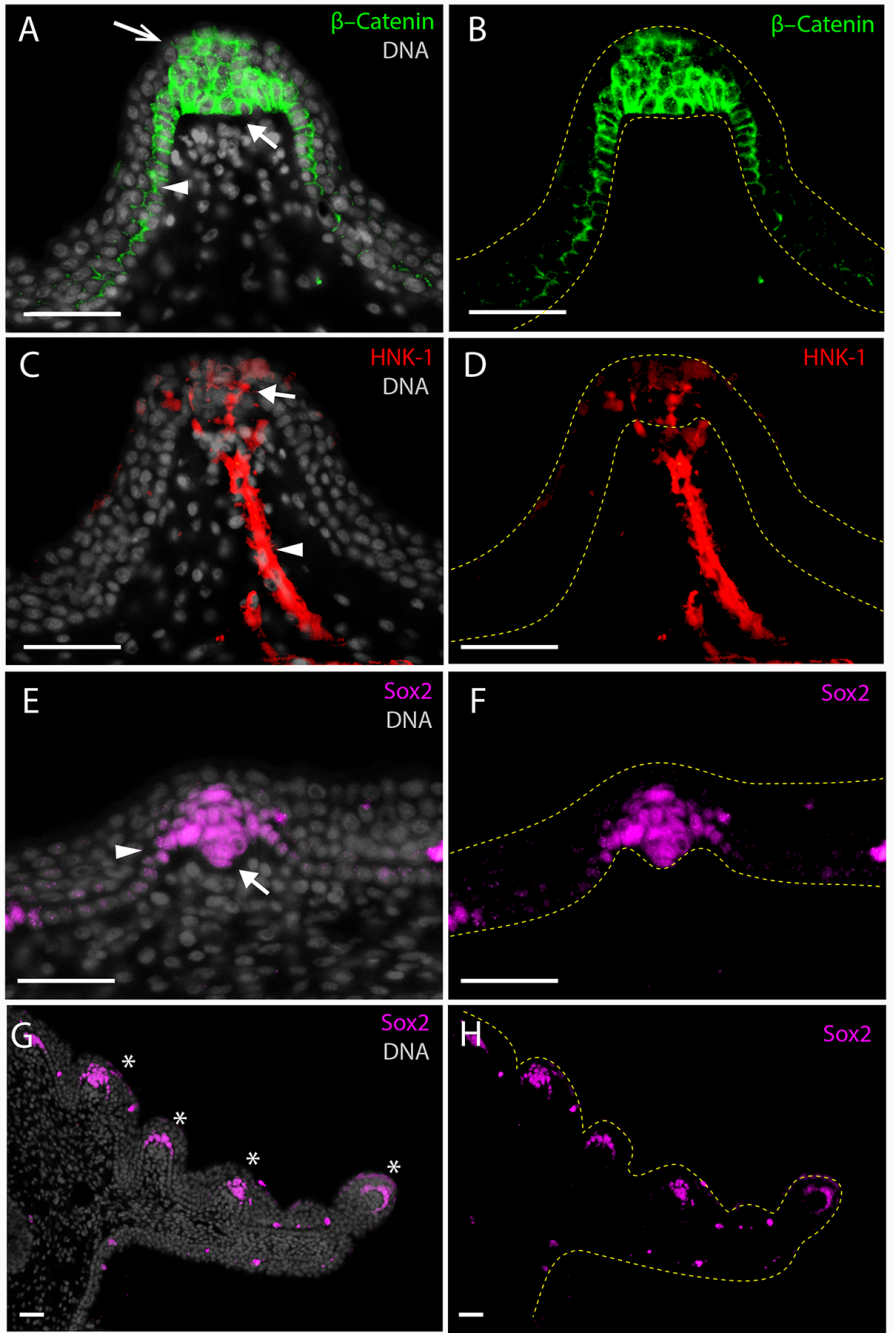
**Immunofluorescent detection of markers of taste bud differentiation in a *Chiloscyllium punctatum* prehatchling stage embryo (TL 103 mm).** (A,B) β-Catenin is concentrated in the cytoplasm and membrane and overlaps the nucleus of all cells of the primordial taste bud ‘bulb’ (closed white arrow), marginal cells (white arrowhead) extending into the basal epithelium of the papilla, as well as on superficial squamous epithelial cells at the apex of the papilla (open white arrow). (C,D) Strong HNK-1 immunoreactivity is detected the length of the afferent nerve fibre innervating the taste papilla (white arrowhead) and within projections extending into the taste bud, and is also detected on cell bodies in the mesenchyme directly underlying the taste bud ‘bulb’ and on cell bodies within the taste bud bulb (1-3 per section) (white arrow). (E-H) Nuclear Sox2 expression is seen in all cells of the taste bud ‘bulb’ including presumptive prospective sensory, support and basal cells (white arrow) as well as within marginal cells (white arrowhead). Sox2 is detected in all definitive taste papillae (white asterisks in G) on the maxillary valve (3-5 per section). DNA is stained with DAPI and shown in grey in all merged images. The borders of the epithelium with the underlying mesenchyme and the oral cavity are delineated with yellow dotted lines in the single colour images. Scale bars denote 50 μm. With the exception of the maxillary (G,H) valves all taste papillae imaged were in a region of the lower jaw between the mandibular valve and the dental lamina.

In order to determine the molecular characteristics of the shark taste papillae and to confidently identify these structural units as functional ‘taste buds’, we investigated the molecular composition of the developing taste bud papillae with range of immunohistochemistry assays. In the embryonic stages of *C. punctatum* we found that Sox2 strongly labels cells within the epithelial core of the differentiating taste bud papilla (Fig. 6). Within the intervening basal oral epithelium adjacent to taste papilla, Sox2 immunolocalisation was low or undetectable. Cells within the developing taste bud ‘bulb’ comprising the presumptive sensory, support and basal cells (Fig. 6A,B) in particular show high levels of Sox2 immunostaining, while expression was notably absent from the overlying stratified squamous epithelial cells. Sox2 was present in all taste buds observed within the oropharyngeal cavity, including the maxillary valves (Fig. 6H), where it distinctly labels multiple taste bud primordia developing on the anterior (labial) surface as well as on the distal tip. However, on the mandibular valve of *C. punctatum* only one focus of Sox2 immunofluorescence, consistent with taste bud primordia development, could be detected at the stage investigated, on the distal tip.

We found that the cell surface antigen HNK-1 immunolocalisation marked the mesenchyme directly underlying the taste unit, axons of afferent nerves innervating the developing taste buds, and a collection of 1-4 presumptive sensory cells within the taste bud bulb (Fig. 6). HNK-1-positive axons enter and terminate within the core of the epithelial bulb of differentiating taste cells, suggesting that already at this stage several taste buds were innervated and may be functional soon after development, or even during the hatching period (Fig. 6). This suggests that sharks may have the capacity to taste even while still in the egg case (at least in oviparous species). In addition, HNK-1 also labelled a number of prospective sensory cells within the taste bud, as well as underlying mesenchymal cells. In *C. puntatum*, β-Catenin accumulates at high levels in all cells of the differentiating taste bud ‘bulb’ as well as within the marginal cells and throughout the basal oral epithelium, albeit at lower levels than within taste bud papillae (Fig. 6). We also noted expression of β-Catenin in a subset of squamous epithelial cells directly overlying the taste bud bulb, in the region of the prospective taste pore opening (Fig. 6).

**Fig. 7.**
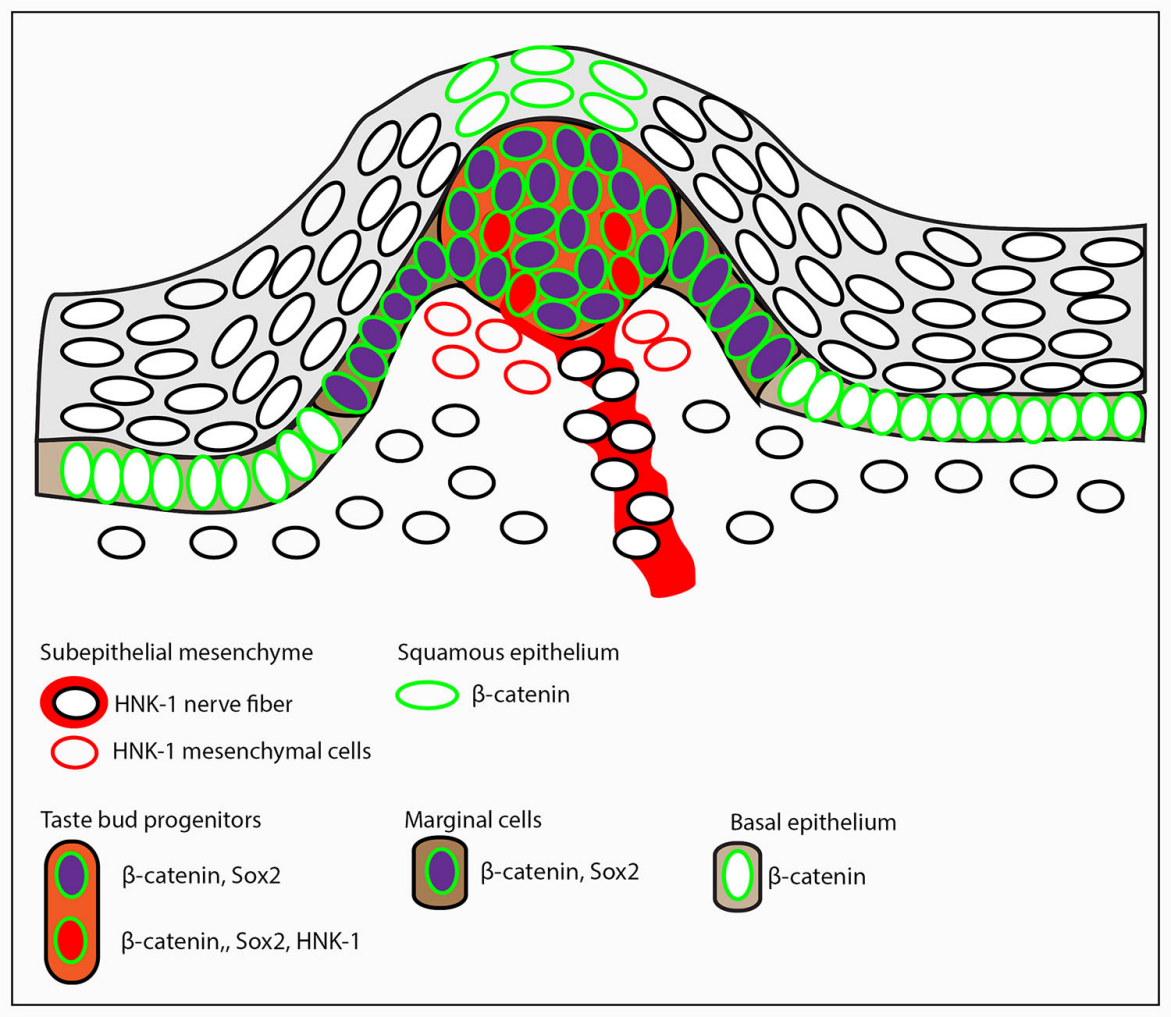
**Model of developing taste bud papilla in prehatchling stage *Chiloscyllium punctatum* embryos.** Cells expressing each of the three markers (Sox2, β-Catenin, HNK-1) of taste papilla development studied are schematically arranged in a model papilla at mid-morphogenesis stage, prior to final differentiation of sensory cells, and opening of the apical pore, but after innervation has occurred. Epithelial contributions to the taste papillae from overlying squamous epithelium (light grey background), and the basal columnar epithelium (light brown background) are denoted as distinct from epithelium derived cells of the progenitors within the taste bud ‘bulb’ (light orange background), and marginal cells (dark brown background).

We present a model summarizing the expression of these immunohistochemical markers of taste papillae development and differentiation (Fig. 7). A number of other immunofluorescent markers, i.e. β-Catenin, label the developing taste bud primordial and the supporting immediately adjacent epithelial cells (Fig. 6). β-Catenin expression overlaps with the expression of Sox2 (Fig. 6) in the terminal epithelial cells, which contain the sensory cells of the taste units.

### Scanning electron microscopy of oral denticles

**Fig. 8.**
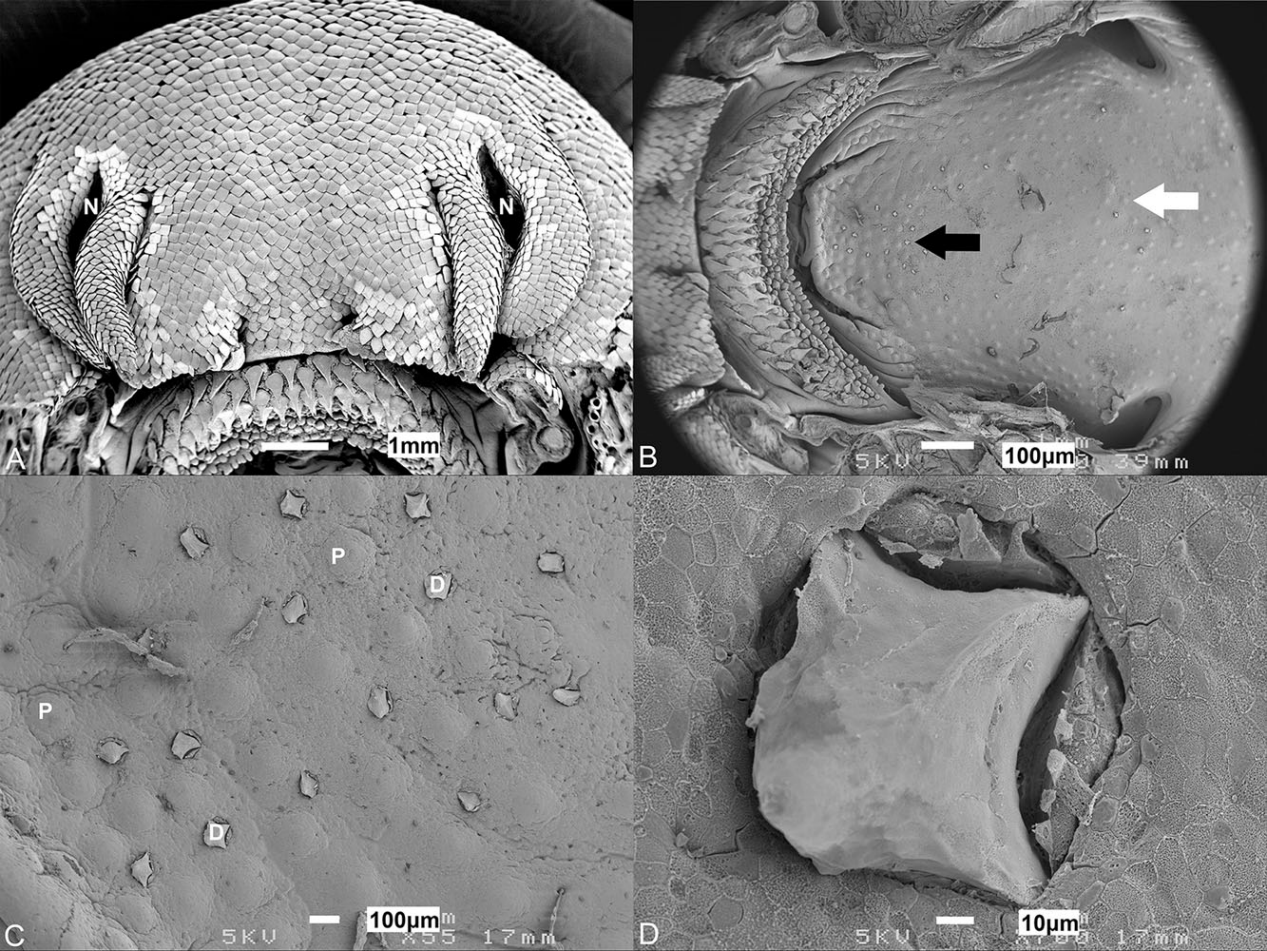
**Scanning electron micrographs of a hatchling *Chiloscyllium punctatum* (TL 192 mm).** (A) External denticles of the rostrum. (B) Dorsal oral cavity showing denticles restricted to the anterior (black arrow) and central oral cavity (white arrow). (C) Higher magnification of these denticles and (D) higher magnification of an individual denticle. N, nares; Sp, spiracle; P, taste papilla; T, teeth; D, oral denticle.

Scanning electron microscopy also revealed denticles, like those of the external shark skin (Fig. 8A), within the oropharyngeal cavity. In embryos less than 134 mm TL, denticles were seen on the external skin but both teeth and oral denticles were absent from the mouth. Embryos between 144 mm and 170 mm TL had some teeth that had broken through the epidermal surface but no oral denticles with the exception of one individual 165 mm TL, which did possess some oral denticles. Statistical analyses of the oral denticle size from all regions of the oropharyngeal cavity revealed that denticle size is highly variable within the one individual. We suggest this large range in size is the result of younger (smaller) denticles replacing older (larger) ones that are being shed. However, larger individuals possess larger denticles. Denticles of the central dorsal oral cavity are more rounded with a greater crown surface area ranging from 15,998±986 μm^2^ (maximal length and width dimensions=181±3 μm and 135±7 μm, TL 503 mm, *n*=5) to 190,485±10,007 μm^2^ (maximal length and width dimensions=457±14 μm and 539±18 μm, TL 1103 mm, *n*=19) to those of the rest of the oropharyngeal cavity. The smallest denticles are generally found on the sides of the pharynx ranging in crown surface area from 12,218±1137 μm^2^ (maximal length and width dimensions=197±10 μm and 102±7 μm, TL 503 mm, *n*=3) to 26,668±2626 μm^2^ (maximal length and width dimensions=218±18 μm and 195±10 μm, TL 844 mm, *n*=3).

**Fig. 9.**
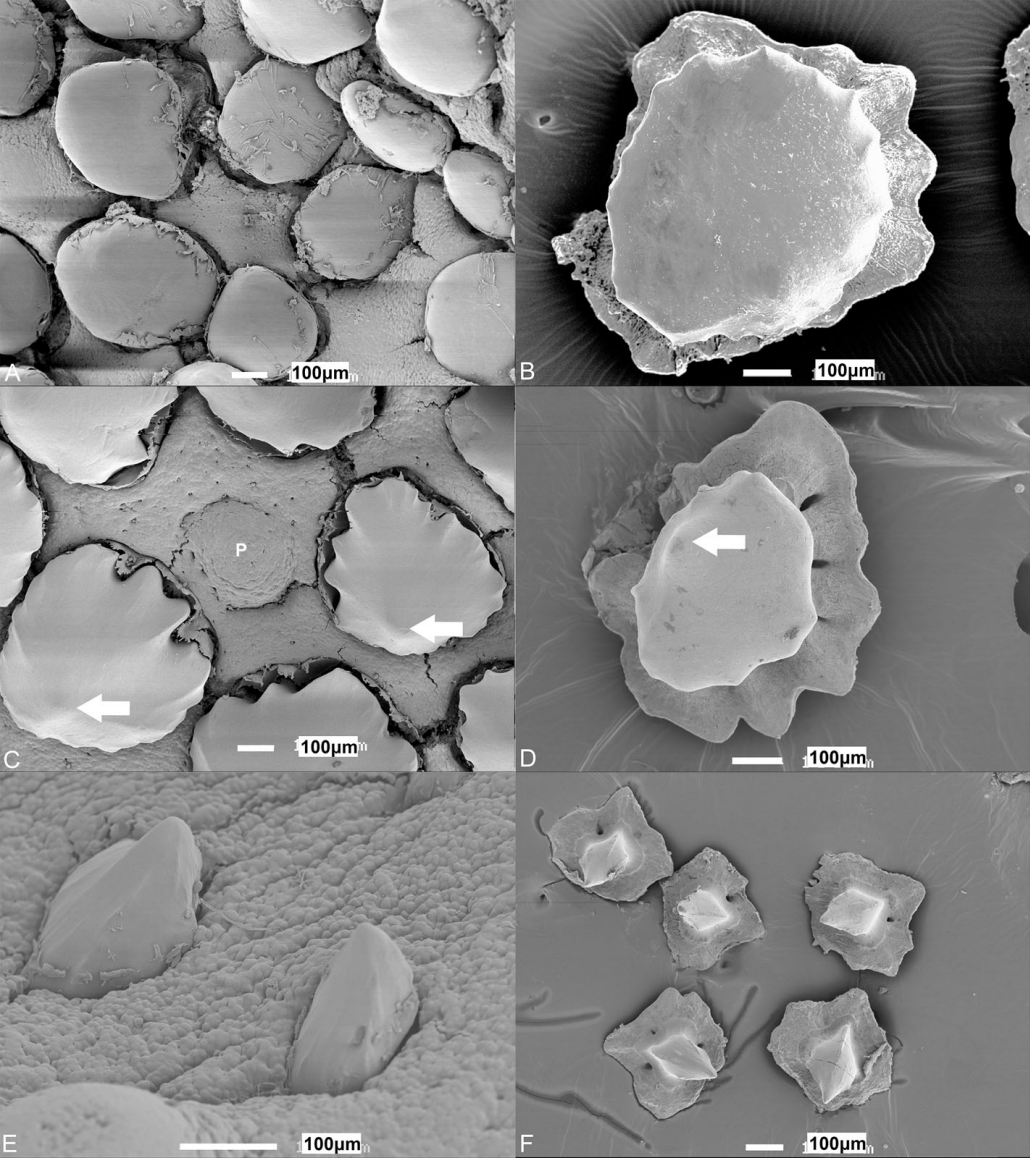
**Morphology of oral denticles.** Scanning electron micrographs of the predominant oral denticles found in the (A) central dorsal oral cavity, which are circular and flat, (C) the central pharynx, which are circular with a raised point on their surface (arrow) and (E) the sides of the oropharyngeal cavity with square/diamond shaped denticles with prominent points on their surface (arrow) of *Chiloscyllium punctatum*. (B,D,F) Extracted denticles from the regions depicted in A,C,E, respectively. P, papilla. TLs for individuals used; (A) 861 mm, (B) 1117 mm, (C) 1103 mm, (D) 601 mm, (E) 635 mm, (F) 503 mm.

The oral denticles in the smallest individuals are square- or diamond-shaped and have an elevated spine protruding into the oral cavity (Fig. 8C,D). In one hatchling of 192 mm total length, the crown surface area of the denticles was 5187±186 μm^2^ (maximal length and width dimensions=86±7 μm and 112±6 μm, *n*=5). These denticles are similar in shape to those seen on the sides of the oropharyngeal regions of immature and mature individuals (Fig. 9E,F). Denticles in the central dorsal oral cavity are circular, large, plate-like structures (Fig. 9A,B), whereas those of the pharyngeal region are similar in silhouette but instead possess a point on their surface, which protrudes into the oropharyngeal cavity like those on the side regions (Fig. 9C,D).

### Distribution of oral denticles

Denticles were absent in all but one embryo (TL 165 mm), where just 28 were located in the central region of the dorsal oral cavity within an area of 0.229 cm^2^. They were only present in the dorsal oral cavity of hatchlings in the central (250±63 per cm^2^), anterior (325 per cm^2^) and side (201±19 per cm^2^) regions (Fig. 8B) and the central region of the ventral oral cavity (75±51 per cm^2^). Denticles covered most of the oropharyngeal epithelium of immature juveniles and mature adults and so only the immature and mature developmental stages were analysed statistically.

Data were square root- and log-transformed before analysis, in order to achieve approximate normality and homogeneity of variances. An ANCOVA was then carried out to determine denticle density differences between regions as the categorical variable and developmental stage of the animal as a covariate. Normality of the residuals was checked using a normal quantile-quantile plot and homogeneity of variances was checked by examining a plot of the residuals versus the fitted values from the model. No significant difference among slopes was found (*F*_9,130_=0.987, *P*=0.454), but there were significant differences between denticle densities of different regions (*F*_9,130_=6.412, *P*≤0.001). Tukey's pairwise comparisons were then performed to determine significant differences. Any differences mentioned are significant at the 5% significance level.

The anterior region of the dorsal oral cavity has more denticles (419±37 per cm^2^) than all regions except for the central region of the dorsal pharynx (408±35 per cm^2^). The central region of the ventral pharynx has more denticles (352±23 per cm^2^) than the ventral oral cavity, and less than the central region of the dorsal pharynx. As no other significant differences are noted, data were pooled to derive a mean density of 300±11 per cm^2^ for the rest of the oropharyngeal regions.

## DISCUSSION

### Taste papillae

Taste is a vital sense for the survival of all vertebrates. In contrast to other vertebrates, taste primordia of fishes including elasmobranchs are located throughout the oropharyngeal cavity from the jaws throughout the pharyngeal cavity to the foregut (Barlow and Klein, 2014). This is in stark contrast to mammals, where taste buds are distinctly localised to specific pockets of epithelia mainly on the tongue. The microvilli, which protrude in separate groups over the apical tip of the papilla, are protrusions of gustatory receptor cells separated by non-microvillus support cells. In *C.* *punctatum*, papillae diameter increases as the animal grows, as does the sensory area of microvilli. This suggests that the taste buds are becoming larger with more receptor and support cells fusing to form the bud. The non-microvillus papillae observed in some specimens may be the result of tissue preparation, damage or represent damaged or aged taste buds that are degenerating, as the cells have a limited lifespan (Beider and Smallman, 1965; Jakubowski and Źuwala, 2001). Papillae without microvilli can also be affected by pollutants prior to capture (Brown et al., 1982; Klaprat et al., 1992). However, gustatory sensitivity compromised by environmental factors is known to reverse and taste responses return back to normal (pre-contaminant) levels (Kasumyan, 1997), which may be due to growth of new taste cells or the regeneration of damaged cells.

Taste buds in a range of teleost fishes are concentrated in areas of food mastication (Fishelson et al., 2004; Linser et al., 1998) and the same is true for *C. punctatum*. When the animals feed they sometimes hold large items in their jaws whilst shaking their head from side to side. While this helps to break up the item, the highest densities of papillae located near the jaws on the oral valves and in the anterior regions of the oral cavity, would also enable taste assessment as the item is held, manipulated and ‘processed’. Animals may often hold an item in the pharynx before it is swallowed so the taste buds of the pharyngeal cavity likely provide the final positive stimulus to ingest the item. This is comparable to teleost fishes, where taste buds are concentrated on ridges and around teeth within the oropharyngeal cavity (Kiyohara et al., 1980; Marui et al., 1983; Komada, 1993; Hara et al., 1994; Linser et al., 1998). As in teleost fishes, the distribution of papillae in *C. punctatum* does not alter with size and subsequent age, although the total number of taste buds present in teleost fishes increases as the animal grows (Gomahr et al., 1992; Komada, 1993; Fishelson and Delarea, 2004). This contrasts the situation in *C. punctatum*, where the total number of papillae is constant (∼1900) and independent of total length and stage of development. However, the total number of papillae in *C. punctatum* is relatively low in comparison with teleost fishes, which can attain totals of between 6600 in the minnow (Kiyohara et al., 1980) and 24,600 in some cardinal fishes (Fishelson et al., 2004).

As the total number of papillae remains constant for *C. punctatum*, the densities within different regions of the mouth decrease as the animal grows. Comparisons with previous studies are therefore difficult as results are dependent on the size of the animal. For *C. punctatum*, the lowest densities range from 420±131 per cm^2^ (mean±s.e.m.) in central regions of the oral cavity in embryos to 8±2 per cm^2^ in the central regions of the pharynx in mature adults. Highest densities found in the anterior regions of the oral cavity range from 941±98 per cm^2^ in embryos to 29±6 per cm^2^ in mature adults. Considerably higher densities occur on the maxillary (3483±286 per cm^2^ for embryos to 111±16 per cm^2^ for mature individuals) and mandibulary (2125±267 per cm^2^ for embryos to 89±10 per cm^2^ for mature individuals) valves. The densities recorded for mature *C. punctatum* are remarkably low compared to teleost species, although this difference may primarily be due to the large size they attain, as hatchlings and embryos have comparable densities. The highest densities of taste papillae recorded for *C. punctatum* are comparable to char, *Salvelinus* sp. (2000-4000 per cm^2^, Hara et al., 1993), rainbow trout, *Oncorhynchus mykiss*, which has densities as high as 3000 per cm^2^ adjacent to the teeth and central ridge of the palate, and 800-1100 per cm^2^ in other areas of the mouth (Marui et al., 1983) and the lowest densities observed in the oral cavity of the minnow, *Pseudorasbora parva* (∼1500 per cm^2^, Kiyohara et al., 1980) and catfish, *Ictalurus natalis* (300-500 per cm^2^, Atema, 1971). Some teleost species however, have considerably higher densities of taste buds, for example the tench, *Tinca tinca* (17,000 per cm^2^, Fishelson et al., 2004) and some cyprinids (∼30,000 taste buds per cm^2^, Gomahr et al., 1992).

The transcription factor Sox2 is a marker of taste buds throughout all stages of development, and is expressed in both progenitor and mature supporting and receptor cells (Okubo et al., 2006, 2009) (Fig. 6A-F) in a variety of vertebrates, including the mouse (Okubo et al., 2006) and zebrafish (Germanà et al., 2009). In mouse, it has been shown that Sox2 expression levels in marginal progenitor cells influences the determination of taste bud sensory versus keratinized papillary cell fates (Okubo et al., 2009). The cell surface antigen HNK-1 is a marker of neural crest and neuronal cells in vertebrates (Bronner-Fraser, 1985). HNK-1 immunolocalisation in *C. puntatum* is consistent with observations in teleost fish (Linser et al., 1998), and rodents, which exhibit HNK-1 immunoreactivity in a stage specific manner in a subset of taste bud cells (Nolte and Martini, 1992). Taste bud sensory cells have a number of properties of neuronal cells, which are unusual for epithelial cells. To untangle potential species-specific differences in taste bud development, which may add to uncertainty about their fundamental origins, further studies in model sharks such as *C. punctatum* will be invaluable as chondrichthyans occupy a basal position compared with other well-studied vertebrates, i.e. mammals, and may help reveal ancestral versus derived mechanisms of taste bud development.

In mouse, activation of the Wnt/β-Catenin signalling pathway is both necessary and sufficient to initiate taste bud formation (Liu et al., 2007) and also plays a role in subsequent differentiation of taste bud primordia (Iwatsuki et al., 2007, Angers and Moon, 2009; Thirumangalathu et al., 2009). Accumulation of β-Catenin to high levels as we observe here is a strong indication that Wnt signalling is active and plays a role in the differentiation and morphogenesis of the taste bud and enveloping papilla in *C. punctatum*. Importantly, we also note that at this stage β-Catenin overlaps all regions of Sox2 expression within the progenitors of the taste bud bulb and marginal cells. The activation of the Wnt/β-Catenin pathway has been shown to be genetically upstream of Sox2 expression in taste buds (Okubo et al., 2006) and it is therefore likely that in *C. punctatum* the Wnt/β-Catenin pathway also has a role to play in regulating Sox2 and therefore determination of taste versus keratinocyte fates. We suggest that taste bud development and patterning in sharks is similar and highly conserved among vertebrates, both in terms of the unit development and molecular characterisation. Our observations of the molecular characterisation of the shark taste papillae allow us to develop a model of taste bud development (Fig. 7) suggesting a highly conserved structural and functional unit that has remained developmentally similar throughout vertebrate evolution. This model will be useful for future studies on the molecular genetic composition of taste in vertebrates. These data highlight remarkable conservation of the mechanism of gustatory development among vertebrates, including sharks.

### Oral denticles

Oral denticles initially protrude through the epidermis of the central region of the dorsal oral cavity around the time of hatching. They then appear to protrude through the epidermis in the central, anterior and side regions of the dorsal oral cavity and the central region of the ventral oral cavity. Immature juveniles and mature adults possess oral denticles throughout the oral and pharyngeal epithelia with the highest concentrations located in the anterior dorsal oral cavity and central regions of the pharynx. When observed feeding, animals appeared to crush prey in their pharynx and, if the items were too large, would hold them in the jaws and shake their heads from side to side. The position of the larger, circular denticles of the central dorsal oral cavity, and the regions of higher density observed in the central pharynx and anterior, dorsal oral cavity, correspond with the parts of the mouth and pharynx that come into contact with the prey most during feeding. As the denticles are oriented so that their spines are facing posteriorly into the rear of the oropharyngeal cavity, it is likely that they help to grip food items during ingestion and direct them posteriorly. We propose that the larger, flatter central denticles are used to prevent abrasion of the mouth lining during food manipulation and consumption, as they provide hard plate-like protection as described for other cartilaginous fishes (Raschi and Tabit, 1992). These suggestions correspond with the findings of Stead (2008) where smaller individuals of *C. punctatum* (<400 mm TL), in which we found no oral denticles, predominantly consumed soft bodied annelid polychaete worms, whereas larger individuals preyed more on teleost fishes presenting the animals with spines and bones and hence a greater need for protection. It is also possible that the ridges on the denticles direct water flow as they do on the external epidermal surface of some elasmobranchs (Reif, 1978; Raschi and Musick, 1984), although it is unknown whether they direct water flow over papillae to aid in gustatory sensitivity or whether they aid in directing water flow over the gills.

Co-localisation of oral denticles and taste buds is a common feature of elasmobranchs (Atkinson and Collin, 2012). These developmentally linked structures must be initiated from the same stock of oral epithelial cells and so must share some elements of a common patterning mechanism. Here, we have shown immunolocalisation of a number of markers of taste bud development and function within the developing taste papillae in *C. punctatum* that co-develops with oral denticles in the oropharyngeal cavity. The co-localisation of these distinct structures in the oropharyngeal cavity supports the study by Atkinson and Collin (2012), which proposes that there is likely restricted territory for taste units. These data therefore offer intriguing evidence that sharks taste prey on biting (‘taste bites’) and, given the concentrated regions of taste papillae associated with the toothed jaws, are able to determine the palatability of the potential prey item from this initial interaction. An important aspect to the maintenance of taste papillae is the ability for regeneration, allowing a specific taste site to remain functional, especially given our observation that taste number changes little across ontogenetic time. Taste papillae are highly regenerative structures that renew throughout the life of the animal (Barlow and Klein, 2014; Perea-Martinez et al., 2013). Our data suggest that taste papillae develop early during development of the shark, and are functional prior to hatching. This early developing and functional gustatory system, coupled with the development of the teeth and protection from the oral denticles, allow the emerging juvenile shark to immediately seek out food *in utero*, at birth or on emergence from the egg case.

## MATERIALS AND METHODS

Specimens of *C. punctatum* were obtained within Queensland, Australia, caught by long-lining in Moreton Bay, or acquired through the captive breeding program at UnderWater World. All stages were observed feeding at UnderWater World; hatchlings (*n*=15) were housed together in small tanks not on display and immature juveniles (*n*=10) and adults (*n*=7) were observed in display aquaria. The hatchlings and immature juveniles were fed by simply depositing pieces of prawns or whitebait into the tanks, whereas adults would predominantly pick up fallen pieces of food from the substrate during shark feeds by divers in the main predator tank, where other elasmobranch species including larger carcharhinids and batoids were present. Animals searched for food under both conditions and were not hand fed.

Animals were anaesthetised with MS 222 (tricaine-methane sulfonate salt 1:250, Sigma) and immediately decapitated anterior to the pectoral fins by severing just posterior to the heart to include all the oral epithelium but minimal oesophageal tissue. All procedures followed the guidelines of the University of Queensland Animal Ethics Committee [AEC Number: ANAT/978/08/ARC (NF)]. Heads were fixed in Karnovsky's fixative (2% paraformaldehyde, 2.5% glutaraldehyde, 2.2% sodium cacodylate, pH 7.4).

Embryos (*n*=4, TL 134-165 mm), hatchlings (*n*=3, TL 175-267 mm), immature juveniles (*n*=12, TL 426-844 mm) and mature adult (*n*=3, TL 1062-1177 mm) heads were cut into dorsal and ventral parts and placed in Toluidine Blue stain overnight to highlight the position of papillae, which stain a darker blue making them more easily distinguished from the surrounding flat epithelia. Dorsal and ventral mouth linings were then photographed with a Sony Cybershot DWC-W200 camera and papillae were counted in various topographic plains using Image Processing and Analysis in Java (ImageJ, NIH) cell_counter.jar plugin before the densities of papillae were calculated. Denticles were counted with the aid of a Nikon SMZ445 dissection microscope and a 0.5 cm^2^ grid dropped randomly five times in each oropharyngeal region. This grid size was chosen, as a limited number of denticles would appear in the window helping to avoid any double counting. The mean for each of these counts (papillae and denticles) was then calculated for each region±standard errors (s.e.m.). ANCOVA and Tukey's pairwise comparison tests were used to determine any significant differences, at the 5% significance level, in papillae and denticle densities and size (papillae diameter and surface area, and maximal width and length dimensions as well as surface area of denticles) during ontogeny.

Pieces of oropharyngeal epithelium were dissected from a range of different-sized individuals of *C. punctatum* (TL 113 mm, 116 mm, 192 mm, 484 mm, 594 mm, 635 mm, 861 mm, 1103 mm) and processed in a Biowave^®^ (PELCO International, CA, USA) for scanning electron microscopy (SEM). Processing involved rinsing in 0.1 M sodium cacodylate buffer in a vacuum at 80 W for 40 s, and postfixing in 1% osmium tetroxide in 0.1 M cacodylate buffer in a vacuum at 80 W for 2 min on, 2 min off, repeated three times. Samples were then progressively dehydrated in an increasing gradient of ethanols at 250 W for 40 s each, and infiltrated with hexamethyldisilazane (HMDS) (1:1 with 100% ethanol, then twice in 100% HMDS) at 250 W for 40 s each, then left to dry overnight. Denticles were extracted from fixed epidermal tissue by macerating dissected epidermal pieces in 0.5 M sodium hydroxide at 80°C for a maximum of 30 min, periodically shaking until the denticles were released. All samples were then platinum-coated (8 nm) in an Eiko IB-5 Ion Coater (Eiko Engineering Company, Japan) and examined using a JEOL JSM 6300F Scanning Electron Microscope (JEOL LTD. Tokyo, Japan). Scanning electron micrographs were examined with ImageJ in order to measure the diameters, surface areas and sensory areas of the papillae and denticles. Please note surface areas are not corrected for three dimensions but instead are representative of the space they occupy when viewed from a position directly overhead.

Tissue samples for light microscopy were embedded in paraffin wax. Processing involved removing the tissue from the fixative and placing it under running water for 15 min. The tissue was then progressively dehydrated in an increasing gradient of ethanols for 45 min each, twice in xylene for 45 min each, wax at 60°C for 45 min, wax at 60°C in a vacuum at 4.08 atmospheres for 45 min (EC 350 Paraffin Embedding Centre, Thermo Fisher Scientific) and then mounted in blocks. Serial sections (4 μm thick) were collected using a Hyrax M25 Rotary Microtome (Carl Zeiss MicroImaging GmbH, Germany), onto glass slides and stained with Haemotoxylin and Eosin or Toluidine Blue.

For molecular characterisation of taste papillae with immunofluorescence, prehatchling stage embryos (*n*=3, TL 88-110 mm) of *C. punctatum* were sourced from the Tropical Marine Centre, Manchester, UK and kept in a marine aquarium in the Department of Animal and Plant Sciences, University of Sheffield, UK. Animals were anesthetized with an overdose of MS222 and decapitated anterior to the pectoral fins. Heads were bisected along the midline and fixation was carried out in freshly prepared 4% paraformaldehyde (PFA) (Sigma) at 4°C in phosphate-buffered saline (PBS, pH 7.4) overnight. Samples were washed in PBS and progressively dehydrated in a gradient of ethanol:PBS solutions and processed for embedding in paraffin wax at the Sheffield University Medical School Department of Infection and Immunity by passing tissue through a series of ethanol, chloroform, and paraffin wax baths according to standard protocols over a period of 24 h. Sections (14 µm) were cut in a sagittal plane using a Leica RM2145 microtome and mounted on Superfrost Ultra Plus slides (Menzel-Gläser) and left to dry on a hotplate at 42°C overnight. Samples were then baked at 58°C for 2 h. Slides were dewaxed in xylene and rehydrated in a decreasing gradient of ethanol:TBS (Tris-buffered saline) solutions. Permeabilisation of cell and nuclear membranes was carried out by 2×10 min washes in TBS-Tween with 0.1% Triton X-100 (Sigma). Heat-mediated antigen retrieval was carried out in a buffer of 0.01 M citric acid with 0.05% Tween-20 (Sigma) at pH 6.0 by preheating the buffer to boiling point, followed by immersion of samples and microwaving for 10 min. Blocking was carried out for 1 h at room temperature in a humidified chamber with 10% foetal goat serum (FGS), 1% bovine serum albumin (BSA) and 0.05% Triton X-100 in TBS at pH 7.6. Primary antibodies were diluted in 1% BSA/0.05% Triton X-100 in TBS pH 7.6 and incubated under a Parafilm strip in a humidified chamber overnight at 4°C. Primary antibodies and concentrations used were as follows: anti-Sox2 (Abcam ab29) 1:250, anti-HNK-1 (DSHB 1C10-s) 1:50, anti-activated β-Catenin (anti-ABC) (Millipore 8E7) 1:250. Samples were washed 2×10 min in TBS-T, and treated with secondary antibodies (1:500 in TBS/1% BSA/0.05% Triton X-100) under Parafilm coverslips in a humidified chamber at room temperature for 1 h. Secondary fluorescently conjugated antibodies used were goat anti-rabbit IgG (H+L) Alexa Fluor 647 (Thermo Fisher A-21245) for detection of Sox2 primary antibody, goat anti-mouse IgG (H+L) Alexa Fluor 488 (Thermo Fisher A-11001) for detection of β-Catenin and goat anti-mouse IgG-CFL 594 (Santa Cruz Biotechnology sc-395766) for detection of HNK-1. Samples were subsequently washed, protected from light, counterstained with DAPI (1:2000 in TBS pH 7.6) (Sigma D9542), postfixed 10 min in 4% PFA:TBS, rinsed in TBS and mounted with ProLong gold antifade mountant (Thermo Fisher P36930). Images were taken on an Olympus BX61 upright epifluorescence microscope with a Hamamatsu Orca monochrome camera and post-processed with Volocity software (v6.3) in the University of Sheffield Wolfson Light Microscopy Facility.
